# IBDTransDB: a manually curated transcriptomic database for inflammatory bowel disease

**DOI:** 10.1093/database/baae026

**Published:** 2024-03-28

**Authors:** Victor Avram, Shweta Yadav, Pranav Sahasrabudhe, Dan Chang, Jing Wang

**Affiliations:** Genomics Research Center, AbbVie Inc, 200 Sidney Street, Cambridge, MA 02139, USA; Genomics Research Center, AbbVie Inc, 200 Sidney Street, Cambridge, MA 02139, USA; Genomics Research Center, AbbVie Inc, 200 Sidney Street, Cambridge, MA 02139, USA; Genomics Research Center, AbbVie Inc, 200 Sidney Street, Cambridge, MA 02139, USA; Genomics Research Center, AbbVie Inc, 200 Sidney Street, Cambridge, MA 02139, USA

## Abstract

Inflammatory Bowel Disease (IBD) therapies are ineffective in at least 40% patients, and transcriptomic datasets have been widely used to reveal the pathogenesis and to identify the novel drug targets for these patients. Although public IBD transcriptomic datasets are available from many web-based tools/databases, due to the unstructured metadata and data description of these public datasets, most of these tools/databases do not allow querying datasets based on multiple keywords (e.g. colon and infliximab). Furthermore, few tools/databases can compare and integrate the datasets from the query results. To fill these gaps, we have developed IBDTransDB (https://abbviegrc.shinyapps.io/ibdtransdb/), a manually curated transcriptomic database for IBD. IBDTransDB includes a manually curated database with 34 transcriptomic datasets (2932 samples, 122 differential comparisons) and a query system supporting 35 keywords from 5 attributes (e.g. tissue and treatment). IBDTransDB also provides three modules for data analyses and integration. IBDExplore allows interactive visualization of differential gene list, pathway enrichment, gene signature and cell deconvolution analyses from a single dataset. IBDCompare supports comparisons of selected genes or pathways from multiple datasets across different conditions. IBDIntegrate performs meta-analysis to prioritize a list of genes/pathways based on user-selected datasets and conditions. Using two case studies related to infliximab treatment, we demonstrated that IBDTransDB provides a unique platform for biologists and clinicians to reveal IBD pathogenesis and identify the novel targets by integrating with other omics data.

**Database URL**: https://abbviegrc.shinyapps.io/ibdtransdb/

## Introduction

Inflammatory bowel disease (IBD) is characterized by chronic relapsing inflammation in the gastrointestinal tract, which consists of two major subtypes, ulcerative colitis (UC) and crohn’s disease (CD) (1). There are 4.9 million cases of IBD worldwide with a steady rise over the last decades (2). Although multiple biologic treatments have been developed to revolutionize the management of IBD [e.g. anti-tumor necrosis factor α (anti-TNFα) and anti-interleukin] (3), there is still a significant unmet need of new targeted therapies for at least 40% inadequate-response patients (4).

With the rapid development of omics technologies in the last decades, transcriptomic datasets have been widely used to understand the pathogenesis of IBD. For example, Wang *et al*. (5) integrated eight tissue transcriptomic datasets from CD and UC patients treated with anti-TNFα therapies with a single-cell RNASeq dataset in UC to reveal the molecular and cellular mechanisms of anti-TNFα inadequate-response patients. Meanwhile, transcriptomic datasets can also integrate with other omics data to identify the novel targets. For example, although novel targets with genetic evidence increases approval by greater than two-fold (6), because of lack of phamacogenetics datasets, it is difficult to identify the novel targets for inadequate-response patients by genetic evidence only. Thus, transcriptomic datasets with treatment response information can be used to prioritize candidate targets identified from genome-wide association studies (GWAS).

Most of transcriptomic datasets have been deposited in the public databases Gene Expression Omnibus (GEO) (7) and ArrayExpress (AE) (8). However, it remains difficult for biologists and clinicians with limited computational knowledge to access and analyze these public datasets. Many web-based tools [e.g. GEOexplorer (9), eVITTA (10), ImaGEO (11) and GREIN (12)] and databases [e.g. Autoimmune Diseases Explorer (13), IAAA (14) and Expression Atlas (15)] have been developed with data visualization, differential gene identification, pathway enrichment analysis or data comparison functions.

However, there are several caveats that hamper the usefulness of these tools for scientific research (Table 1). First, because of the unstructured metadata and data description of datasets in GEO and AE, most of web-based tools/databases can only search the datasets by the original data ID or disease (9–11), which makes it difficult for users to find datasets of interest (e.g. data of certain treatment). Expression Atlas allows users to input multiple keywords but cannot return the accurate comparisons due to lack of the intersection search capability. For example, if searching ulcerative and infliximab for TNF on the Expression Atlas, ‘Crohn’s disease vs control’ comparison is listed in the result with the highest log2(fold change). Second, because of the lack of an effective query system, most of web-based tools/databases do not support multiple dataset comparisons or perform meta-analysis to integrate multiple datasets, e.g. validating one target in multiple anti-TNFα datasets or comparing and summarizing the treatment effect across different conditions. Even if eVITTA and ImaGEO provide some of these functions, users still need to manually find their datasets of interest based on data ID. Third, all web-based tools provide functions to identify the differentially expressed genes, but most of them do not support comparisons based on multiple conditions [e.g. pre-treatment responders vs after-treatment responders in the CD colon samples from GSE16879 dataset (16)]. Although some tools [e.g. GEOexplorer (9)] allow users to manually select samples for the comparisons, this is not practical or feasible for studies with large sample sizes. Fourth, none of existing tools/databases supports cell type deconvolution analysis. Though single-cell RNASeq data provide an unprecedented opportunity to improve the diagnosis and treatment of auto-immune diseases (17), very few single-cell RNASeq data contain treatment and response status information. Thus, it is difficult to identify the association between cell types and treatment effects using this more advanced technology. Cell deconvolution method (18) can address this problem by estimating cell fractions of bulk transcriptomic dataset based on single-cell RNASeq data. However, none of existing tools/databases enable users to perform this type of analysis.

**Table 1.** Features unique to IBDTransDB or shared with other tools

	IBDTransDB	GEOexplorer	ImaGEO	eVITTA	GREIN	Expression Atlas	Autoimmune diseases explorer	IAAA
Type	Database	Tool	Tool	Tool	Tool	Database	Database	Database
**Database**								
Manually curated data sets	∏	X	X	X	X	X	X	X
Support data sets from GEO and AE	∏	Only GEO	Only GEO	Only GEO	Only GEO RNASeq data	∏	Only GEO	Only GEO
Data query system	Intersection search based on five attributes	Query by GEO ID	Query by GEO ID	Query by GEO ID	Not support intersection search	Not support intersection search	Query by GEO ID and disease	X
**Data visualization**								
Data summary	∏	∏	X	∏	∏	X	X	X
Differential gene	∏	∏	X	∏	∏	∏	∏	∏
Gene/gene list exploration	∏	X	X	Only gene	X	X	∏	∏
Signature analysis	∏	X	X	X	X	X	X	∏
Enrichment analysis	∏	∏	X	∏	∏	X	Only KEGG	X
Cell deconvolution	∏	X	X	X	X	X	X	X
**Data comparison**								
Gene-level	∏	X	X	∏	X	∏	X	X
Pathway-level	∏	X	X	∏	X	X	X	X
**Data integration**								
Gene-level meta-analysis	∏	X	∏	X	X	X	∏	X
Pathway-level meta-analysis	∏	X	X	X	X	X	X	X

To address these challenges in the transcriptome analyses, we propose IBDTransDB (https://abbviegrc.shinyapps.io/ibdtransdb/), a manually curated transcriptomic database for IBD. IBDTransDB has five key features: (i) a manually curated database with 34 high-quality transcriptomic datasets (2932 samples) and 122 differential gene lists based on comparisons from multiple conditions; (ii) a query system supporting 35 keywords from five attributes; (iii) IBDExplore: interactive visualization of differential gene expression, pathway enrichment, gene signature and cell deconvolution results; (iv) IBDCompare: data set comparisons across different conditions based on selected genes or pathways; (v) IBDIntegrate: meta-analysis to prioritize a list of genes/pathways based on user-selected data sets and comparisons. We also provided two case studies to demonstrate the utility of this unique resource in IBD pathogenesis identification and target identification by integrating with other omics data.

## Materials and methods

### Dataset collection and metadata curation

**Figure 1.**
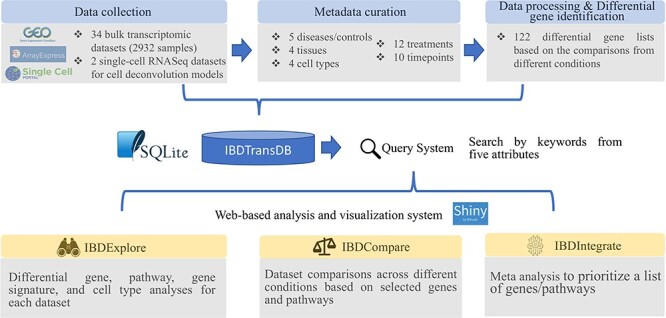
IBDTransDB for exploratory, comparative and integrative datasets to identify and validate the novel IBD targets. Top panel: data processing and meta data curation for 34 IBD bulk transcriptomics datasets and 2 single-cell RNASeq datasets. Bottom panel: three data analysis modules.

A total of 34 human IBD bulk transcriptomic data matrices and metadata were downloaded from GEOquery (19) (microarray datasets in GEO), GREIN (12) [RNA sequencing (RNA-Seq) datasets in GEO] or ArrayExpress (Figure 1). All data matrices had been quality controlled and normalized by the authors of the respective original publications (microarray datasets) or GREIN (RNA-Seq datasets). For microarray datasets, probe IDs provided by each dataset were mapped to HUGO gene symbols by biomaRt R package (20). If multiple probe IDs were mapped to one HUGO gene symbol, these IDs were collapsed by calculating the median expression of IDs in each sample. The metadata were manually curated based on 35 keywords we defined from 5 attributes: 5 diseases/controls, 12 treatments, 10 timepoints, 4 tissues and 4 cell types (Supplementary Table S1). Some of these keywords were mapped to multiple ontologies [e.g. MedDRA LLT (Medical Dictionary for Regulatory Activities Lowest Level Term) (21), MeSH terms (Medical Subject Headings) (22) and NCIT (National Cancer Institute Thesaurus) (23)]. Among 34 datasets, there are 5 CD PBMC datasets, 11 CD tissue datasets, 10 UC tissue datasets and 8 tissue datasets with both CD and UC patient samples. Furthermore, 16 datasets include treatment samples from different timepoints, and 20 datasets had control samples. The details of 34 datasets are listed in Supplementary Table S2. Differentially expressed genes from 122 comparisons were pre-calculated by limma R package (24) (Supplementary Table S3). All bulk datasets, metadata and comparison results were stored in an SQLite database (version 3.37.0).

CD PBMC single-cell RNASeq data matrices and meta data (25) were downloaded from GEO (GSE134809) while UC single-cell RNASeq datasets (26) were downloaded from broad single cell portal (https://singlecell.broadinstitute.org/single_cell). All single-cell datasets were processed by Seurat analysis pipeline (27).

### Data analysis modules

IBDTransDB has three analysis modules: IBDExplore, IBDCompare and IBDIntegrate (Figure 1).

### IBDExplore module

IBDExplore module allows accurate data selection based on the query from five attributes (e.g. infliximab treated CD datasets from colon biopsies). After a data set of interest is selected, five functions are available for users to explore the results.

- ‘Dataset Description’ function shows the data description and PCA (Principal Component Analysis) plot colored by the selected conditions. The *P*-value between different conditions for the selected PC can be calculated by Wilcoxon rank-sum test.
- ‘DGE Viewer’ function visualizes the differentially expressed genes of a selected comparison in a table and volcano plot. Genes selected by users in the table can be highlighted in the volcano plot, and their expression difference between conditions is visualized with the boxplots. The *P*-value from the pre-calculated output with limma will be shown in the boxplot.
- ‘Signature Viewer’ function calculates the mean expression of a user-input gene list in each sample as a sample-specific signature score. Then, the signature scores of samples from different conditions are plotted in a boxplot. The *P*-values among conditions are calculated by Wilcoxon rank-sum test.
- ‘Enrichment Analysis’ function performs either ORA (over-representation analysis) or GSEA (gene set enrichment analysis) analysis for a selected differential gene list based on the 18 databases (Supplementary Table S4) provided by the WebGestaltR package (28). Multiple interactive plots provided by WebGestaltR are imbedded in IBDExplore for visualization of enrichment results (e.g. bar plot and volcano plot for result summary, venn diagram and GSEA enrichment plot for individual pathway).
- ‘Cell Deconvolution’ function estimates the cell fractions of bulk transcriptomics data using a deep learning model developed and trained with CD PBMC or UC single-cell RNASeq dataset (25, 26). CD PBMC model is used to deconvolve the blood bulk samples while UC tissue model is used for bulk tissue samples. The detailed information about model generation can be found in Wang *et al*. (5) and Menden *et al*. (18). Briefly, 50 000 pseudo-bulk samples were generated for the single-cell RNASeq dataset. Then, we built a four-layer deep neural network model with L1 as the loss of function and Rectified Linear Unit (ReLU) activation for all layers except the last layer and softmax activation for the last layer. The model was trained and validated based on the leave-one-out subject method. A *P*-value comparing the cell fractions between conditions is calculated by Wilcoxon rank-sum test.

### IBDCompare module

IBDCompare module enables easy comparison of gene/pathway significance in multiple comparisons from the same datasets (e.g. gene significance between non-responder and responder at baseline vs at Week 6 in one infliximab-treated dataset) or the different datasets (e.g. gene significance between non-responder and responder at baseline in all infliximab-treated datasets). Gene significance was from pre-calculated limma output, while significance of 311 KEGG (29) and 1414 Reactome (30) pathways in 122 comparisons was pre-calculated based on GSEA analysis from WebGestaltR. After inputting interesting genes/pathways and selecting datasets based on the five attributes, a comparison table is used to visualize the gene/pathway significance in all comparisons from each selected dataset. Grid of boxplots are also available to visualize the gene expression difference between conditions from at most six comparisons.

### IBDIntegrate module

IBDIntegrate module performs the meta-analysis for a set of genes/pathways based on the selected comparisons and ranks these genes/pathways by meta *P*-values. For gene-level meta-analysis, two-sided *P*-value of each gene from limma output is converted to two one-sided *P*-values based on the directionality of log2(fold change).


$$Upregulated{\ }p = \begin{cases}
{p/2{\ }} & {if{\ }log2\left( {fold{\ }change} \right) > 0}\\
{1 - p/2}&{otherwise{\mathrm{ }}}
\end{cases}$$

$$Downregulated{\ }p = 1 - upregulated{\ }p$$

A meta *P*-value for the up-regulated *P*-values or down-regulated *P*-values of a gene in all selected comparisons is calculated by the R package ‘poolr’ (https://cran.r-project.org/web/packages/poolr/index.html). The final meta *P*-value is the minimum between meta up-regulated *P*-value and meta down-regulated *P*-value. Adjusted meta *P*-value is calculated based on the Benjamini-Hochberg method.

For pathway-level meta-analysis, one-sided *P*-values of each KEGG or Reactome pathway is based on the *P*-value and normalized enrichment score from GSEA analysis. Meta *P*-value calculation is the same with gene-level meta-analysis.

## Results

Two case studies related to infliximab treatment were used to demonstrate the unique features of IBDTransDB for revealing IBD pathogenesis and identifying the novel targets by integrating with other omics data.

### Understanding the infliximab resistance molecular and cellular mechanisms

**Figure 2.**
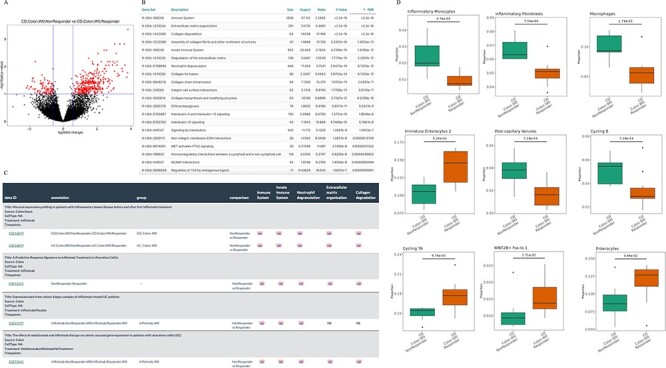
Understanding the infliximab resistance molecular and cellular mechanisms. (A) Volcano plot of differentially expressed genes between non-responders and responders at baseline in GSE16879 CD colon samples. (B) Top 20 enriched Reactome pathways based on the up-regulated genes from (A). (C) Comparison of five representative Reactome-enriched pathways from (B) in five non-responder vs responder comparisons from four infliximab-treated IBD datasets. (D) Significant cell types between non-responders and responders at baseline in GSE16879 CD colon samples.

Improving the understanding of molecular and cellular mechanisms that facilitate the differential response of anti-TNFα therapy can accelerate discovery and development of novel targets for these non-responders. To test the analytical pipeline of IBDTransDB for mechanism identification, we reanalyzed the infliximab-treated GSE16879 CD colon samples (16). With a filter of |log2(fold change)|> 0.58 and FDR < 0.1, 363 and 94 genes were up- and down-regulated in non-responders compared with responders at baseline, respectively (Figure 2A). Based on the ‘Enrichment Analysis’ function in the IBDExplore module, 32 Reactome pathways were enriched with up-regulated genes (FDR <0.05) but no pathway was related to down-regulated genes. Many immune pathways (e.g. innate immune system and neutrophil degranulation) and tissue remodeling pathways (e.g. extracellular matrix organization and collagen formation) were found to be related to the non-responders, which was consistent with previous study (5) (top 20 pathways were shown in Figure 2B). To identify whether these pathways were also significant across multiple infliximab treatment datasets, four datasets were selected in IBDCompare based on the following keywords: disease keywords (‘CD’ and ‘UC’), tissue keyword (‘Colon’), treatment keyword (‘Infliximab’) and timepoint (‘W0’), which included one CD comparisons and four UC comparisons between non-responders and responders at baseline. Five representative pathways were significant in almost all five comparisons (*P*-value <0.05 and NES >1), which indicated that CD and UC had the similar infliximab non-response molecular mechanisms (Figure 2C).

Cell fractions of samples from non-responder and responder baseline groups were estimated by the deep learning model generated by a UC single-cell RNASeq data with 51 cell types (26) (‘Cell deconvolution’ function in the IBDExplore module) and nine cell types had significant difference between non-responders and responders (*P*-value <0.05 and minimum of cell fractions in each group >0.5%, Figure 2D). Cell types with higher fractions in non-responders were inflammatory monocytes, inflammatory fibroblasts, macrophages, post-capillary venules and cycling B cells, which were highly consistent with Reactome enrichment results and can cross-validate molecular and cellular mechanisms. Together, these demonstrate IBDTransDB’s capacity for mechanistic identification and validation.

### Integrating IBD GWAS genes with transcriptomic datasets to prioritize candidate targets for infliximab non-responders

**Figure 3.**
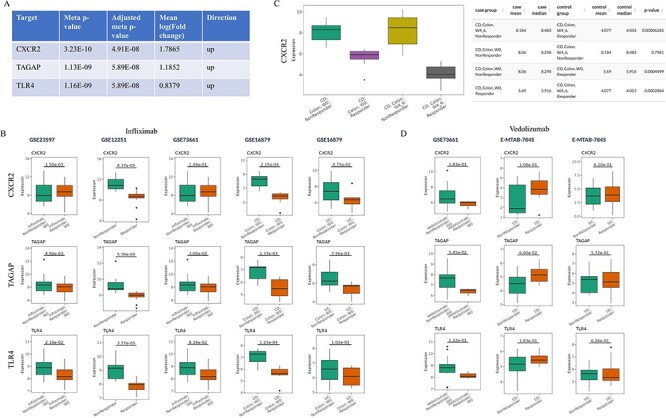
Prioritization of the candidate targets with genetic evidence for the treatment of infliximab non-responders. (A) Top three candidates with adjusted meta *P*-value <0.05 and |mean log2(fold change)|>0.58 from IBDIntegrate module. (B) Comparison of three candidates in five non-responder vs responder comparisons from four infliximab-treated IBD datasets. (C) Comparison of CXCR2 expression between after-treatment and baseline samples for responders and non-responders in GSE16879. (D) Comparison of three candidates in three non-responder vs responder comparisons from two vedolizumab-treated IBD datasets.

Barrio-Hernandez *et al*. identified 152 IBD GWAS genes based on L2G score >0.5 (31), which were used to perform meta-analysis in the IBDIntegrate module. Because CD and UC patients shared the similar anti-TNFα non-response mechanisms, the same five comparisons with the previous case were selected from four datasets. Under adjusted meta *P*-value <0.05 and |mean log2(fold change)|>0.58, 31 genes were identified as candidates that had consistently higher or lower expression in non-responders than responders (Supplementary Table S5 and Figure 3A). Selecting these comparisons in the IBDCompare module can visualize the expression distribution of these genes in different comparisons (Figure 3B). CXCR2 was the most significant one and Lyu *et al*. (32) suggested that infliximab failure in UC patients could be treated by inhibiting CXCR2. Some datasets [e.g. GSE16879 (16)] also included the infliximab post-treatment samples that can be used to validate the candidates by comparing multiple conditions in the ‘Signature Viewer’ function of the IBDExplore module. For example, CXCR2 expression was significantly decreased after infliximab treatment in responders but not in non-responders (Figure 3C).

IBDCompare module can also compare the candidates across different treatments. For example, vedolizumab was another FDA-approved treatment for IBD patients (33). However, top three candidates (CXCR2, TAGAP and TLR4) had no difference between non-responders and responders at baseline for both CD and UC patients and thus may not be the candidate targets for the treatment of vedolizumab non-responders (Figure 3D). Overall, this exemplifies the utility of IBDTransDB in prioritizing and validating a list of candidates with genetic evidence based on multiple datasets.

## Discussion

Transcriptomic data have been widely used for IBD to reveal the disease mechanisms and combine with other omics data to identify novel targets. However, there is still a lack of a well-curated and consistently annotated database for large-scale data reuse. Here, we present IBDTransDB, a manually curated transcriptomic database with comprehensive query system, interactive data visualization, comparison and integration. IBDTransDB has the following unique features compared to the existing web-based transcriptomic data visualization and analysis tools or databases (Table 1). First, IBDTransDB includes 34 manually curated bulk transcriptomic data with 2932 samples from GEO and AE and provides the comprehensive data query system based on 35 keywords from 5 attributes. IBDTransDB also includes 122 pre-calculated comparisons. Second, IBDExplore provides cell deconvolution model to estimate the cell fractions of bulk transcriptomic data based on single-cell RNASeq data. Integrating cell fractions with treatment information can help identify the novel cellular targets and therapeutic strategies such as cell reprogramming and cell therapy. Third, IBDCompare allows users to compare genes/pathways across multiple datasets within or across datasets, which can be used for the target validation or indication extension. Fourth, IBDIntegrate can rank a list of genes/pathways by performing the meta-analysis based on the selected datasets and comparisons.

With comprehensive data query, visualization, comparison and integration across different diseases and treatments, IBDTransDB represents a convenient and powerful exploratory, comparative and integrative tool for scientists to analyze IBD transcriptomic datasets. One limitation of IBDTransDB is users cannot upload, analyze and compare their own IBD transcriptomic datasets in three modules. This is mainly because most of vocabularies in the users’ metadata may not be included in IBDTransDB keyword table (Supplementary Table S3) and it is difficult to harmonize them with the current database automatically. We plan to develop a data uploader to guide users to prepare and upload data files in the future. Meanwhile, we will continue to incorporate new datasets as well as novel functions to enhance the IBDTransDB database.

## Supplementary Material

baae026_Supp
